# Integrating Deep Learning-Based IoT and Fog Computing with Software-Defined Networking for Detecting Weapons in Video Surveillance Systems

**DOI:** 10.3390/s22145075

**Published:** 2022-07-06

**Authors:** Cherine Fathy, Sherine Nagy Saleh

**Affiliations:** Computer Engineering Department, College of Engineering and Technology, Arab Academy for Science and Technology (AAST), Alexandria 1029, Egypt; sherine_nagi@aast.edu

**Keywords:** fog/edge computing, Internet of Things (IoT), Software-Defined Network (SDN), Software-Defined IoT, video surveillance, weapon detection, deep-learning, YOLOv5n

## Abstract

Due to the widespread proliferation of multimedia traffic resulting from Internet of Things (IoT) applications and the increased use of remote multimedia-based applications, as a consequence of COVID-19, there is an urgent need to develop intelligent adaptive techniques that improve the Quality of Service (QoS) perceived by end-users. In this work, we investigate the integration of deep learning techniques with Software-Defined Network (SDN) architecture to support delay-sensitive applications in IoT environments. Weapon detection in real-time video surveillance applications is deployed as our case study upon which multiple deep learning-based models are trained and evaluated for detection using precision, recall, and mean absolute precision. The deep learning model with the highest performance is then deployed within a proposed artificial intelligence model at the edge to extract the first detected video frames containing weapons for quick transmission to authorities, thus helping in the early detection and prevention of different kinds of crimes, and at the same time decreasing the bandwidth requirements by offloading the communication network from massive traffic transmission. Performance improvement is achieved in terms of delay, throughput, and bandwidth requirements by dynamically programming the network to provide different QoS based on the type of offered traffic and current traffic load, and based on the destination of the traffic. Performance evaluation of the proposed model was carried out using the mininet emulator, which revealed improvement of up to 75.0% in terms of average throughput, up to 14.7% in terms of mean jitter, and up to 32.5% in terms of packet loss.

## 1. Introduction

In the last decade, specifically the past two years, the COVID-19 pandemic resulted in a drastic increase in multimedia traffic (video and audio) transmission through networks. According to Cisco 2021, Global Networking Trends Report [1], an average of 4.7 times more employees are now working from home compared to before the pandemic, which led to 62% of companies deploying video conferencing applications. As a result, Information Technology (IT) is facing a new set of challenges for supporting remote workers, which include security across a more distributed computing landscape, end-user behavior, application performance, and IT operations.

Moreover, Cisco Annual Internet Report (2018–2023) [2] anticipates that the Machine-To-Machine (M2M) connections’ share will increase from 33% to 50% from 2018 to 2023. This is due to an increasing number of M2M applications, which impacts the growth of devices and connections. Within the M2M connections category (which is also referred to as the Internet of Things (IoT)), connected home applications will represent nearly 50% of the total M2M connections by 2023, which implies a significant demand for bandwidth in the future connected home applications.

According to a recent report published by Allied Market Research [3], the global video surveillance market was valued at $42.94 billion in 2019 and was expected to reach $144.85 billion by 2027, registering a Compound Annual Growth Rate (CAGR) of 14.6% from 2020 to 2027. This will result from the increasing demand for safety in high-risk areas, the integration of IoT in surveillance cameras, and the increasing demand to monitor the COVID-19 cluster in high-risk areas. Moreover, in the Internet of Multimedia Things (IoMT), smart surveillance systems play a significant role in smart cities due to their capabilities in automated human and object recognition; tracking and taking account of risk factors; in enhancing Intelligent Transportation Systems (ITS); and in supporting smart health, with real-time video monitoring of patients [4].

Several articles [5,6,7] have discussed the problem of weapon detection in surveillance videos and how it is very important to automate such a process, since it will reduce the huge efforts needed to manually review the video streaming, reduce the violation of privacy, and most importantly provide a very fast response that may lead to crime prevention. Finding important scenes in video surveillance is a very important aspect when aiming to trigger certain events according to the content of such frames. The severity of an action detected by a surveillance camera could result in the need for immediate intervention from one or more services such as the police or a hospital.

Detecting hidden weapons has been previously addressed in [8] by the use of active electromagnetic signals detected using a walk-through metal detector and a sensor array. Previous to their research, weapon detection systems based on metal detectors mainly detected the presence of large metal objects, required setting an adjustable threshold to identify elements of threat, and were affected by the human body. Article [8] managed to reconstruct an image from the measured electromagnetic signals and use it for weapon detection. The advantage of this system when compared to ours is the ability to detect concealed weapons, yet it is limited by the existence of expensive hardware. Our proposed system is based on camera-based systems which are more commonly used now, as mentioned by [3], as they are a crucial component of smart surveillance.

Article [9] provided a comprehensive review of automatic pistol and knife detection in different computer vision-based systems. The article discussed the benefits of creating automatic weapon detection systems and mentioned some of its challenges. For instance, the lengths of the detected weapons differ according to the parameters of the imaging systems, since the position of the camera to the target weapon is not fixed. Furthermore, the high variation in the types, colors, and shapes of the weapons needs to be addressed by all systems and mainly are dependent on the variation of weapons available in the training datasets. Finally, the viewing angle of the weapon could greatly affect the recognition ability of any system.

Object detection and recognition is a domain that is being tackled in deep learning research for the past few years [10]. Several challenges have been presented when trying to detect objects in an image, for instance, the huge difference in the appearance of each target object class from one scene to another. Another challenge is the balance between the speed and accuracy of a detection algorithm, especially on edge devices. In surveillance applications, it is very important to have a fast detection rate and, since the application is critical, the accuracy of such a system is very important.

YOLO [11] is an object detection algorithm that was initially presented in 2016 and, since then, YOLO’s first version along with its successors have been employed in a variety of applications such as vehicle recognition [12] and face recognition [13] for its fast and accurate detection. The latest release of YOLO, namely YOLOv5, has been proposed by [14] and presented as several models of different sizes. YOLOv5 architecture comprises three stages: backbone, neck, and head. The backbone is the feature extraction stage which utilizes the CSPDarknet model, the neck stage fuses the extracted features using the PANet model, and finally, the head which comprises a YOLO layer that generates the detection parameters [15].

Article [6] has compared different object detection techniques based on both sliding-window and region-based methods. Experiments were conducted on a dataset collected by the researchers and not provided for public use. The results of the analysis have shown that YOLOv4 has produced the highest mean average precision and F1-Score. The authors discussed that real-time detection analysis required the existence of a real-time dataset which is currently still not available.

Video surveillance, as explained before, plays a significant role in smart cities, whether in safety or tracking of COVID-19 cases. However, it is a bandwidth-hungry application. Video surveillance systems generate multimedia traffic that is considered delay-sensitive traffic, especially in the case of early detection of crime and the attempt to prevent the crime or in the case of the fast rescue of victims. Network support for this type of traffic is required to ensure the QoS requirements of the application in terms of bandwidth, delay, and jitter.

Once a weapon is detected in surveillance videos, there is a need for dynamic multimedia traffic management techniques that support the requirements of this type of traffic in terms of delay and bandwidth. Moreover, to cope with the highly dynamic nature of networks, whether the Internet or the IoT, it is required to adopt architectures/paradigms that can be reprogrammed to match current network conditions. The Software-defined Networking (SDN) paradigm has been referred to as a promising technique in recent network management research studies.

SDN is a network paradigm that separates the network’s control logic (control plane) from the traffic-forwarding routers and switches (the data plane). As a result of this separation, network switches become simple forwarding devices, and control logic is implemented in a logically-centralized controller, simplifying policy enforcement, network (re)configuration, and evolution [16]. The centralized global network view, programmability, flexible management, and separation of the data plane and control plane are the key benefits of using SDN [17]. Deploying SDN for decentralized IoT network provisioning and management is critical. The OpenFlow protocol is a standard Controller-Data Plane Interface (C-DPI) that allows controllers and data plane devices to communicate.

Several articles [4,18,19,20] present different proposals to deploy SDN in video surveillance systems; however, performance evaluation results of their proposed platforms either were not provided or insufficient.

Table 1 reviews SDN-based surveillance systems highlighting the artificial intelligence (AI) and SDN roles in these systems. In [21], filtering videos at the back-end server already consumes bandwidth as the filtering module takes in n input video streams and outputs a subset of k streams to be displayed to the monitoring person. Moreover, the authors did not provide any details about computer vision techniques used in filtering videos at the back-end server and did not provide any performance evaluation results, especially regarding delays.

Moreover, edge computing solves resource-constrained problems by getting computation near the edge of IoT devices. The distribution of edge nodes across the network overcomes the delay and the centralized computation challenges found in the IoT. New edge technologies classify and filter IoT big data generated from an increased number of connected devices before transmitting it to the central cloud data center, which alleviates the challenge of traffic overload and privacy concerns [23]. Article [24] highlights the challenges for edge computing and proposes SDN as a solution for these challenges. Since the SDN paradigm depends on a centralized software-based controller, it will relieve simpler edge devices from executing complex networking activities. Moreover, Ref. [24] explains the reasons behind the emergence of edge computing, namely: real-time QoS, delay sensitiveness, battery lifetime, the regulation of core network traffic, and scalability. Also, Ref. [25] review the fog computing and SDN solutions to overcome the IoT’s main challenges.

QoS is typically defined as an ability of a network to provide the required services for selected network traffic. The main aim of QoS is to give priority with respect to QoS parameters including, but not limited to: bandwidth, delay, jitter, and loss [17]. The integrated service (IntServ) model and the differentiated service (DiffServ) model are considered to be the conventional methods for QoS. In the case of SDN, QoS models are implemented by queues and meters in OpenFlow switches.

Article [17] provided a review of the QoS capabilities of the OpenFlow protocol through its different versions. Moreover, they introduced seven categories in which QoS can benefit from the concept of SDN, namely: multimedia flows routing mechanisms, inter-domain routing mechanisms, resource reservation mechanisms, queue management and scheduling mechanisms, Quality of Experience (QoE)-aware mechanisms, network monitoring mechanisms, and other QoS-centric mechanisms. Furthermore, they highlighted the benefits of using the SDN paradigm in ensuring QoS. These benefits can be summarized as follows:

1. SDN controller has a global view of the whole network.
2. Set of flow policies and classes are unrestricted while it is limited in conventional networks because of many vendor-specific firmware at use.
3. Through the use of an SDN controller, network statistics can be monitored on different levels with respect to per-flow, per-port, and per-device while overcoming conventional network’s limited global view and QoS possibilities, and per-hop decision making.

Motivated by the increase of IoT multimedia traffic generated from surveillance systems, our objective in this research is to develop an intelligent adaptive architecture that ensures QoS over the best-effort network by deploying AI techniques at the edge to decrease the bandwidth requirement of these systems and by leveraging the SDN paradigm to reprogram the allocation of available bandwidth among traffic flows based on the global view of network conditions (made available at the SDN controller through communication with forwarding devices over OpenFlow protocol).

The contribution of this article is as follows:

1. The article investigates the deployment of different paradigms to support real-time video surveillance application that is considered one of the key applications in smart cities. It proposes the application of deep learning models at the edge, as an exploration of the edge computing paradigm. The proposed deep learning model employs the most recent lightweight version of YOLO to manage real-time surveillance and detection of weapons. This YOLO version was chosen after extensive experiments over several YOLO versions with different parameters.
2. Moreover, network support for video surveillance applications was introduced by deploying a software-defined networking paradigm (SDN) to play the role of the network core, to control bandwidth allocation among different traffic flows to speed up crime prevention upon weapon detection achieved through AI models implemented at the edge.
3. In addition, an investigation is carried out through simulation to justify that the integration of AI techniques with software-defined networking paradigms fulfills IoT-based multimedia application constraints in terms of delay and bandwidth.
4. Finally, it provides recommendations and directions for future work in the domain of using AI and SDN in the IoT-based surveillance systems in smart cities.

The rest of the article is organized as follows: Section 2 presents a detailed explanation of our intelligent and adaptive QoS framework after giving a brief background on OpenFlow protocol. Section 3 highlights the simulation results of our proposed framework. We summarize the findings of the article in the concluding section.

## 2. Materials and Methods

In this section, our proposed model architecture is described. First, a background on specific related points of OpenFlow protocol is explained. Then, the proposed model architecture which includes the IoT device layer, edge computing device layer including the proposed deep learning model, SDN core network, and the adaptive QoS algorithm is presented.

### 2.1. OpenFlow Required Background

An OpenFlow Switch, such as Open vSwitch (OVS), consists of one or more flow tables and a group table, which conduct packet lookups and forwarding, and an OpenFlow channel to connect with an external controller. Each flow table in the switch comprises a collection of flow entries, each of which consists of match fields, counters, and a set of instructions to be implemented on matching packets. The received packet on the input port is filtered based on packet header fields in a flow table, and a set of actions is executed on the matching packets. These actions include rate-limiting using a meter table, forwarding using a group table, forwarding to the relevant output port, or dropping the packet. For non-matching packets, the packets can be passed to the controller over the OpenFlow channel, or they can be dropped.

The controller deploys the OpenFlow protocol (the south-bound interface of the SDN controller) to add, update, and delete flow, group, and meter entries from flow, group, and meter tables, respectively. Same-tables manipulation can be carried out using applications running on the north-bound interface of an SDN controller. The controller uses three flow-rule installation modes, namely: proactive mode, reactive mode, and hybrid mode [23].

A meter table is made up of meter entries that define per-flow meters. In its instruction set, a flow entry can define a meter that monitors and controls the rate of the aggregate of all flow entries to which it is connected. Arriving packets are forwarded to a meter as specified by a matching flow table entry as explained in [19].

### 2.2. Proposed Model Architecture

The general architecture of Software-Defined Internet of Things-Edge (SDIoT-Edge) comprises three planes including SDN data, control, and an application plane. The data plane is made up of resource-constrained IoT devices that use edge cloudlets to offload heavy compute operations. The OpenFlow protocol is used to communicate with the control plane. Furthermore, the control plane communicates with the application plane using REST APIs as presented in [23].

**Figure 1:**
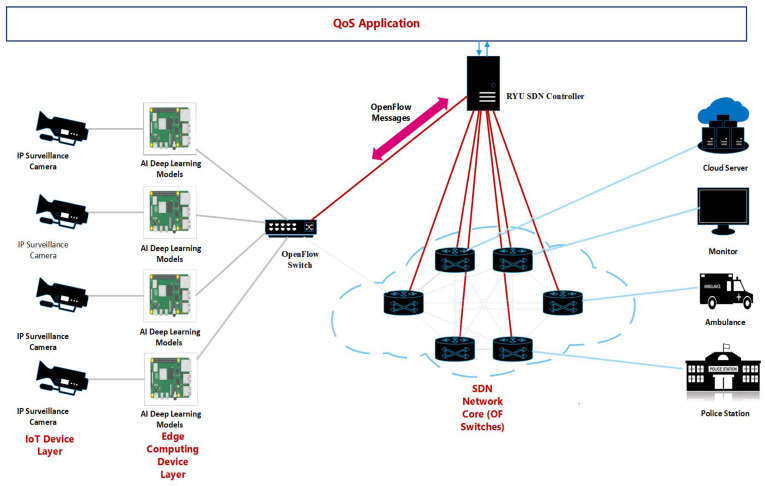
Proposed model architecture.

Our proposed model is depicted in Figure 1. Our proposed model is composed of four main components: IoT device layer, edge computing device layer, SDN core network, and QoS application. The IP surveillance cameras (IoT Device Layer) generate a continuous video stream of the monitored area which is then fed into a raspberry PI implementing the deep learning AI models (edge computing device). The output of the raspberry PI is a small video file which is a group of frames containing the weapon detected. This video file is routed through SDN OpenFlow switches that implement the proposed adaptive QoS algorithm. The OpenFlow switch classifies the traffic based on the destination port number and allocates bandwidth to different classes of traffic based on the meter table installed by the RYU controller in each switch. The meter table settings give priority to video files containing weapons to ensure fast delivery through the OpenFlow switches to authorities to help in the fast rescue of victims and to help in the prevention of crimes. The proposed QoS module is adaptive as it collects free bandwidth available in each class and reassigns it to the hungry class with priority to video traffic that ensures lower packet drop and lower jitter. The small video file represented a very small percentage of the original video file collected continuously from IP surveillance cameras. This filtration process at the edge reduces the massive bandwidth requirement in case of sending the whole video to a monitoring person or authorities.

The IoT device layer and edge computing device layer together form the IoT smart node. The proposed architecture supports a different level of QoS requirements for traffic classes by applying classification and prioritization of flows. The architecture depends on the use of OpenFlow meters to manage bandwidth allocation for individual flows where meter rates are dynamic and adaptive to match current network conditions in terms of available bandwidth and offered type of traffic. Our target is supporting multimedia streaming applications. We assume that all switches are OpenFlow switches and are connected to a centralized controller through OpenFlow protocol. Flows are classified into several classes of QoS and Best-effort (BE). Initially, each class is allocated a bandwidth share. The following subsection explains the several components of our proposed architecture and gives justification for our design choices.

#### 2.2.1. IoT Device Layer

The IoT device layer consists of IP surveillance cameras that continuously generate a video stream of the monitored area which is then fed into the edge computing device layer that implements the AI models.

#### 2.2.2. Edge Computing Device Layer

**Figure 2:**
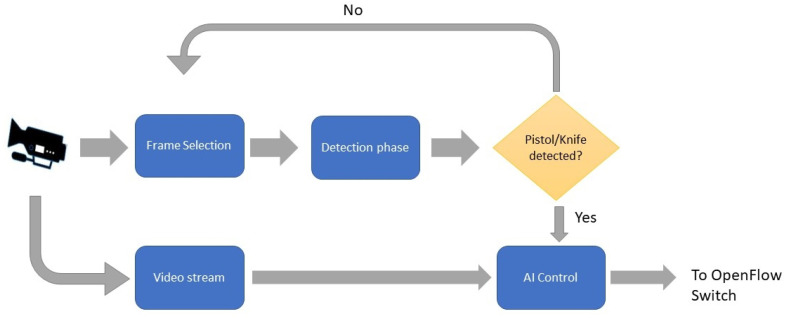
Proposed deep learning based weapon detection model.

The edge computing device layer is responsible for applying the proposed AI deep learning models, thus assuring a fast, responsive system. The proposed AI model is presented in Figure 2, which aims at detecting the threat in the video surveillance stream and sending a small portion of the video over to the OpenFlow switch. The video stream captured by the surveillance camera is continuously input to the frame selection module. The chosen frames are then input to the detection phase to check them for the presence of weapons. Once a weapon is detected, a signal is sent to the AI control such that the frame with the detected weapon and a subsequent set of frames are then sent to the OpenFlow switch. The details of each block of the proposed model will be further explained in the upcoming paragraphs.

##### Dataset

Article [7] discussed the problem of discriminating between small objects appearing in surveillance videos and claimed that, when analyzing results produced in previous research, high precision resulted from detecting larger-sized objects than those small-sized ones given the same dataset and model.

**Figure 3:**
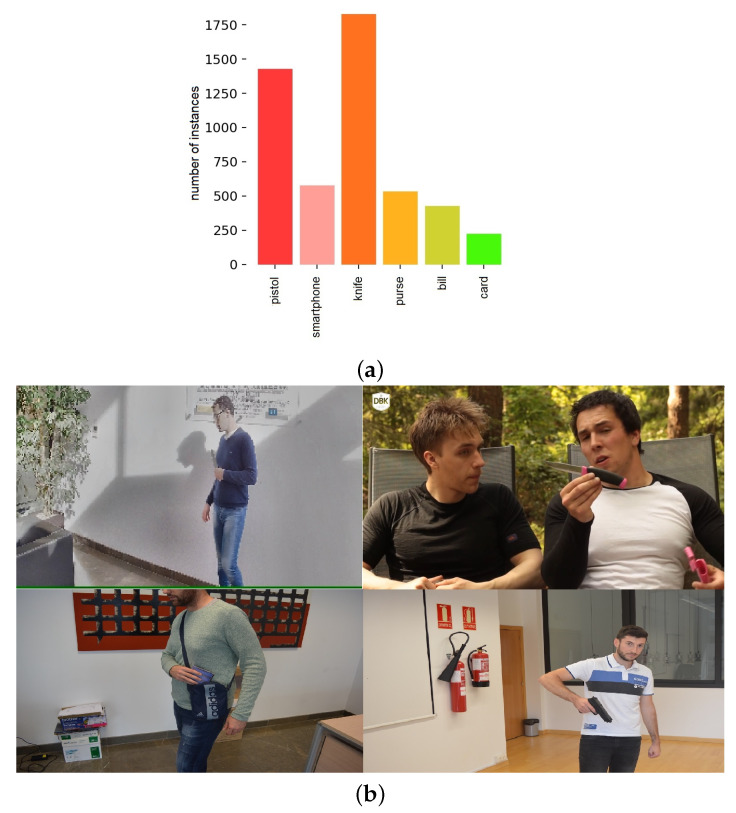
SOHAs_weapons dataset. (**a**) Class distribution of training dataset. (**b**) Sample images from the dataset.

To address this problem, they built an image dataset, namely the Sohas_weapon dataset, including six of the most common small object classes: pistol, knife, smartphone, bill, purse, and card. The dataset included images collected from previous datasets along with ones taken by cameras of different qualities and resolutions. The images have a variety of scenes, some of a close appearance to the camera and others from a faraway angle. The dataset has the multiclass distribution shown in Figure 3a. It can be deduced from the figure that the six classes do not have an equal distribution, thus making the learning process challenging, especially for the smaller classes. Samples from the training dataset are shown in Figure 3b. The Sohas_weapon training dataset is used to train different YOLOv5 object detection models, each for 200 epochs, and tested using the preset test data. A comparison between the models and the results will be presented in the following section and, accordingly, the choice of the best model for detection in our real-time surveillance system will be deduced.

##### Frame Selection and Object Detection

Since the proposed model is based on a real-time surveillance application that detects the presence of weapons, it is critical to choose an object detection model that has both high accuracy and detection rate. Most object detection methods are known to be computationally expensive, therefore the proposed AI model had to be built using a lightweight detector to best accommodate the real-time system. In the proposed model, several experiments were conducted to assess versions of YOLOv5, as will be shown in the upcoming section, that was designed by [14] to suit the needs of edge computing, namely, the YOLOv5n. Benchmark comparisons of YOLOv5 versions have shown that it outperformed its predecessors and other famous object detectors. Furthermore, our experiments included two more lightweight updates to YOLOv5 that were presented by [26] as YOLOv5-lite e and YOLOv5-lite s. Both lightweight versions were proposed as updates to the YOLOv5 by adding a shuffle channel and removing the focus layer and some of the slice operations, thus resulting in a faster, easy-to-deploy model with an acceptable accuracy.

When analyzing the proposed deep learning model, the time taken by the detection phase to analyze a single frame is the aspect upon which the keyframes to be converted are chosen. Therefore, the speed of the detection phase has a direct influence on the number of keyframes that could be extracted every second from the video stream. Further analysis will be illustrated in the following section of the models addressed, and the suitable number of keyframes selected per second will be deducted.

##### Control

Each chosen frame is assessed as to whether or not a weapon is detected. If no weapons exist then the frame selection from the video stream and weapon detection process continues. On the other hand, in case a weapon is detected, the current frame is sent to the AI control with a bounding box of the detected weapon, and the current frame, along with a subsequent number of frames retrieved from the video stream, is then sent to the OpenFlow switch.

#### 2.2.3. SDN Core Network

The SDN network is composed of a set of interconnected OpenFlow switches. The switches communicate with the RYU controller with the OpenFlow protocol. The RYU controller was chosen for the following reasons [27]:

- It contains a separate module for QoS.
- RYU extensively supports OVSDB protocol through a library using which REST applications could be built to configure the database of OVS.
- RYU was implemented in Python programming language, which facilitated quick prototyping to design our intelligent QoS framework.
- Since RYU supports the OVSDB configuration protocol, it is suitable for our open-source software switch i.e., Open vSwitch (OVS) used within our framework.

Our developed application explores the meters entity defined in the OpenFlow protocol, starting from version 1.3, to dynamically control the allocation of available bandwidth between different traffic flows. In our work, we define three classes: class 1 for video traffic, class 2 for VoIP traffic, and class 3 is best-effort traffic. Initially, we define the minimum required bandwidth for each class as a percentage of the available bandwidth, so that the total bandwidth required of the three classes equals the defined bandwidth of links, giving initially higher weight to class 1 (video traffic flow). However, we proved through simulation, that even in the case that class 1 was not given higher weight in the initial bandwidth allocation phase of the algorithm, it will soon collect the free bandwidth available from the remaining classes, as class 1 has higher priority in a collection of free bandwidth. The incoming flow is classified into one of the three predefined classes based on the destination port number. Once the topology starts, OpenFlow switches communicate with the controller and get registered. At that time, the controller sends a message to install the meter table for each OpenFlow switch as instructed by the QoS application. Upon the arrival of a packet, the packet is classified based on the destination port number and is assigned the meter id corresponding to its class. Then, the flow is inserted in the flow table with the meter id added to the action field. Traffic exceeding the meter-configured bandwidth will be dropped. The operation of the QoS application is explained in Algorithms 1–3.
**Algorithm 1** Adaptive Quality of Service Algorithm: Initialization Phasei ← Number of traffic flow classes**For each** class of traffic flow i **do**Allocate bandwidth $ABW_{i}=$
$w_{i}$ · bw: $\sum_{k=1}^{k=i}w_{k}.bw$= bw∀ OpenFlow Switches **do** Install meter tableStart S seconds countdown timer (Mtimer) for meter table monitoring module

**Algorithm 2** Adaptive Quality of Service Algorithm: Operation Phase**For each** Packet In **do**Classify traffic flow & assign meter-id for that flow based on Destination Port NumberInsert flow in flowtable with meter id in the action field

**Algorithm 3** Adaptive Quality of Service Algorithm: Adaptation Phase**if** Mtimer expires **then**∀ switches doCollects meter table statistics (used bandwidth, free bandwidth, dropped)**if** adaptation timer expires **then** ∀ meter m ∈{meters}**if** Required bandwidth > allocated bandwidth + safety margin**then** add m to set of hungry meters**if** Required bandwidth < allocated bandwidth + safety margin**then** calculate free bandwidth for this meterReconfigure meter table by reassigning free bandwidth to hungry meters based on traffic class priority

## 3. Results and Discussion

### 3.1. Evaluation of Proposed AI Model

The evaluation of the proposed AI model is crucial to the assessment of the proposed system as it will directly affect its applicability to real-time video surveillance. This subsection will first discuss the evaluation metrics of the deep learning-based models then the results obtained will be presented and discussed.

#### 3.1.1. Object Detection Evaluation Metrics

To evaluate the performance of the object detection model, several metrics were applied namely: precision (*P*), recall (*R*), and mean average precision (mAP) [28]. Precision indicates the percentage of the true positive (TP) predictions from all the labels predicted as positive (whether True (TP) or False (FP)). The equation is given by

(1) $$P=\frac{TP}{TP+FP}.$$

Recall measures the model’s capability of predicting the positive label. Thus, it calculates the percentage of the TP from all those images actually labeled as positive (whether True (TP) or False (FN)) following the equation:

(2) $$R=\frac{TP}{TP+FN}.$$

Furthermore, the mAP metric is one of the most important metrics when assessing object detection models. The mAP is the mean of the average precisions calculated for each class given a specific threshold. The threshold sets the value for the intersection over union (IoU) for the prediction. If the IoU is higher than the threshold then the classification is counted as a true positive. Otherwise it is classified as a false positive. If an object is not detected, then it is counted as a false negative.

#### 3.1.2. Object Detection Results

Using the SOHAs weapon training dataset, three lightweight versions of YOLOv5 were trained each for 200 epochs, to assess their performance. The three trained models were then tested using the 857 test images and the results are as shown in Table 2. For each model, the precision, recall, and mAP at a threshold value of 0.5 are reported and it can be seen that the YOLOv5n model has resulted in the highest mAP in almost all the classes, specifically the pistol and knife classes which represent the critical classes for our application.

Table 3 shows the number of parameters of the three lightweight models and shows that YOLOv5-lite s and YOLOv5n are of almost similar size. Article [10] presented a comparison of different lightweight object detectors and has shown that the most recent models such as MobileNetv3, OFA, and MobileViT-S need to train 5.4, 7.7, and 5.6 million parameters respectively. Since the application is real-time surveillance, therefore the number of parameters will have a direct effect on the inference time. Article [26] has reported that the YOLOv5-lite s were tested on the Raspberrypi 4B and resulted in an average of 84ms per frame. Accordingly, the frame rate processed in this application could not exceed 10 frames per second when applying the selected model on the Raspberrypi 4B.

**Figure 4:**
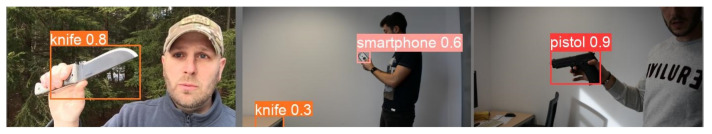
Sample of objects detected by YOLOv5n with their confidence.

**Figure 5:**
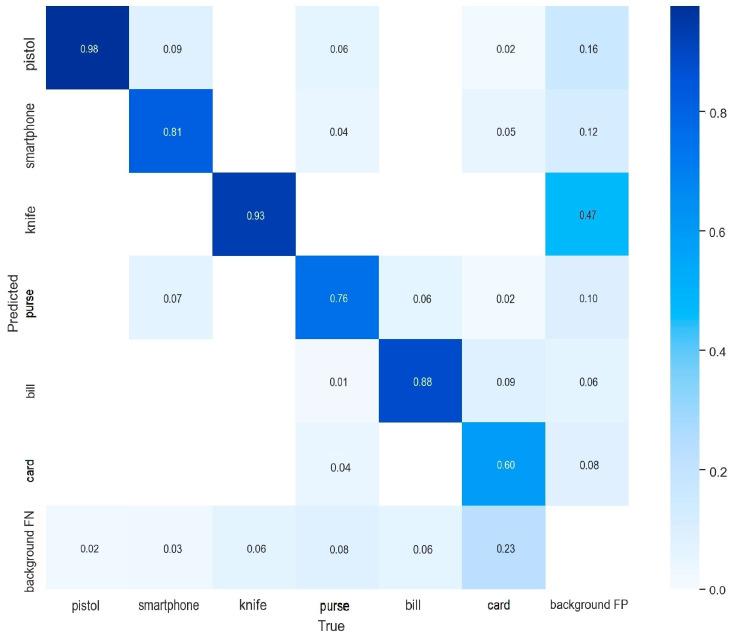
Confusion matrix showing the results distribution among all class when employing YOLOv5n model.

Figure 4 shows a sample of the output of the testing results. The image on the left shows a true positive detection of a knife with a confidence of 0.8, and the image on the right shows a true positive detection of a pistol with a confidence of 0.9. The centered image shows a false negative detection of the edge of the table as a knife and a true positive detection of a smartphone. The confusion matrix of the test results is also presented in Figure 5, showing the diagonal as all the correct classifications and the other cells presenting the misclassified samples either as other classes, or false detections, or not discovered.

### 3.2. Adaptive QoS Framework Evaluation

In this subsection, we present and discuss the experimental results of our adaptive SDN-based QoS framework. Our adaptive QoS framework was evaluated using Mininet software [29]. Mininet is an open-source network emulator that permits users to build virtual software-defined networks composed of an OpenFlow controller, a network of multiple OpenFlow-based Ethernet switches, and multiple hosts connected to those switches. The SDN controller involved in the emulated platform is the open-source RYU for the reasons discussed previously. In addition, the iPerf tool is deployed. For UDP traffic, using iPerf, a client can create UDP streams of specified bandwidth and can measure packet loss, delay, and jitter. The VLC software is deployed to perform the video streaming. Wireshark software is running in the background to capture packets of different traffic flows for performance metric calculation and analysis.

#### 3.2.1. Performance Metrics

Three performance metrics were used to evaluate our proposed SDN-based adaptive QoS framework, namely: mean jitter, packet loss, and average throughput.

##### Jitter

*Jitter* is defined as a variation in the delay of received packets as shown in Equation (3), where $D_{j}$ represents the delay experienced by the *j*-th packet. The difference in transit time between two consecutive packets of the tagged flow can be written as

(3) $$J\left(j\right)=D_{j+1}-D_{j},$$

The average end-to-end delay jitter is then given by the expected absolute value of this random variable as in Equation (4)

(4) $$J=E[|D_{j+1}-D_{j}|],$$

#### 3.2.2. Scenario 1

**Figure 6:**
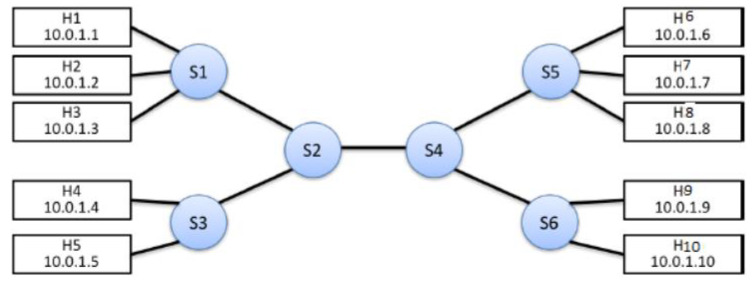
Network setup used in evaluation.

The set of experimental results was conducted over the network setup illustrated in Figure 6, and using software packages presented in Table 4. The first set of experiments compares two cases, namely: the case of applying an adaptive QoS framework, and the case of not applying any QoS techniques. In this scenario, a topology consisting of ten hosts and six OpenFlow switches was built, and the initialization phase is carried out as illustrated in Table 5. In Table 5, the initial bandwidth allocated to each traffic class is carried out by configuring three meters with three different bands on each OpenFlow switch. As explained before, each meter is allocated a percentage of the available bandwidth. Afterward, four traffic flows were generated as follows:

1. A VoIP server runs on port 6000 on H7, and best-effort (BE) server runs on port 7000 on H10 using iperf commands as follows: h7.cmd(’iperf -u -s -p 6000 -i 10 > h7cls2_Server.log &’); h10.cmd(’iperf -u -s -p 7000 -i 10 > h10be_server.log &’)
2. A four-parallel VoIP client streams run on H4 and BE client runs on H3 using iperf commands as follows: h4.cmd(’iperf -u -c 10.0.1.7 -p 6000 -P 4 -b 3M -i 10 -t 120 > h4_iperf_cls2_client.log &’); h3 cmd(’iperf -u -c 10.0.1.10 -p 7000 -b 5M -i 10 -t 120 > h3_iperf_be_client.log &’)
3. Start two parallel video streaming clients over RTP on H2 and H1 each required BW 1 Mbps using VLC commands
4. Start RTP servers on H8 and H9 which listen on Port 5004 using VLC commands

**Figure 7:**
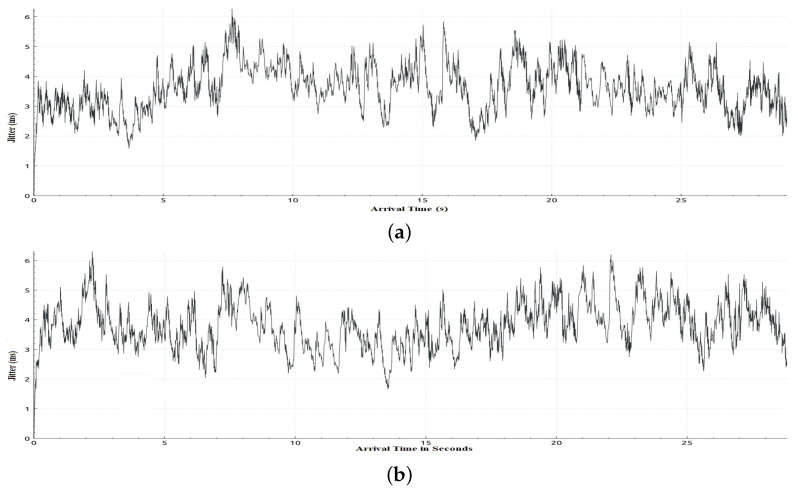
Jitter obtained in Scenario 1 in case of applying and without applying the proposed adaptive QoS model for 30-seconds video streaming traffic flow (**a**) Jitter obtained in Scenario 1 in case of applying the proposed adaptive QoS model, (**b**) Jitter obtained in Scenario 1 without applying the proposed adaptive QoS model.

**Figure 8:**
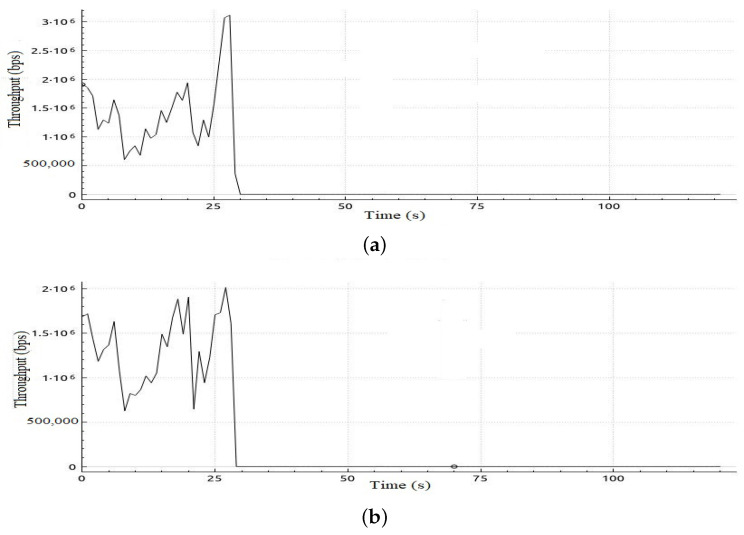
Throughput obtained in Scenario 1 in case of applying and without applying the proposed adaptive QoS model for 30-seconds video streaming traffic flow (**a**) Throughput obtained in Scenario 1 in case of applying proposed model, (**b**) Throughput obtained in Scenario 1 without applying the proposed adaptive QoS model.

**Figure 9:**
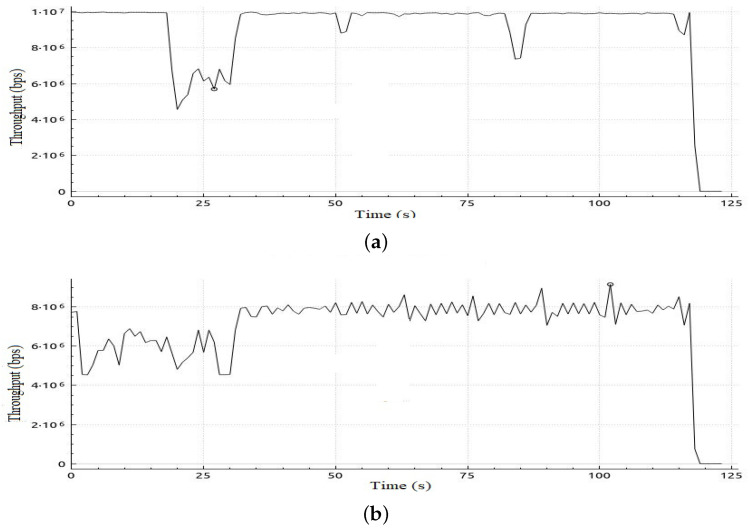
Throughput obtained in Scenario 1 in case of applying and without applying proposed adaptive QoS model for 120-seconds VoIP traffic flow (**a**) Throughput obtained in Scenario 1 in case of applying the proposed adaptive QoS model, (**b**) Throughput obtained in Scenario 1 without applying the proposed adaptive QoS model.

**Figure 10:**
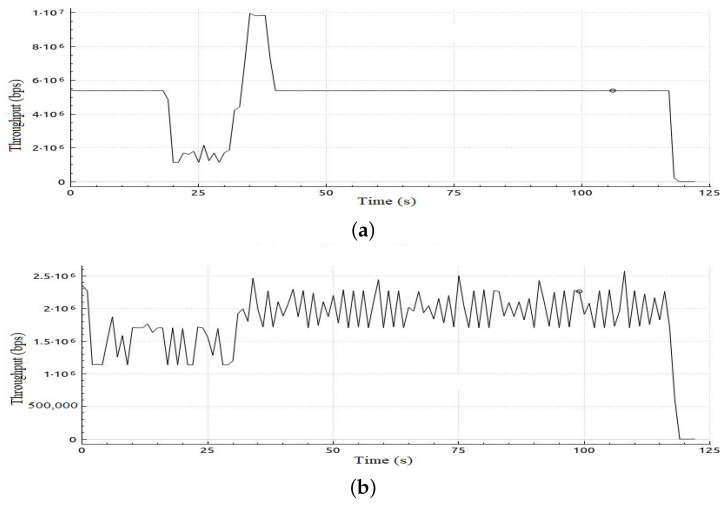
Throughput obtained in Scenario 1 in case of applying and without applying the proposed adaptive QoS model for 120-s BE traffic flow (**a**) Throughput obtained in Scenario 1 in case of applying the proposed adaptive QoS model, (**b**) Throughput obtained in Scenario 1 without applying the proposed adaptive QoS model.

Table 6 shows sample calculations for the adaptation phase for Scenario 1. The free bandwidth of meter 1 is reassigned to meter 2 even if meter 2 and meter 3 are both hungry for bandwidth. However, meter 2 has a priority according to our priority strategy that gives priority to class 2 traffic over class 3 traffic. Later on in the simulation, when class 2 is not in the hungry-for-bandwidth list, free bandwidth can be allocated to class 3, i.e., BE traffic flow. In Table 7, a comparison of the results of the mean jitter and number of lost packets for video streaming traffic flows in both cases, with and without applying the proposed adaptive QoS framework, is displayed. As depicted in Table 7, our proposed adaptive QoS framework decreases the number of lost packets by an average of 7.85% for video streaming traffic flows and improves the mean jitter for video streaming traffic flows by 8.4%. Figure 7 presents the jitter results for video streaming traffic flow. Improvement of 22% in average throughput for VoIP traffic and 75% in average throughput for BE traffic is illustrated in Table 8. In addition, as illustrated in Figure 8, throughput reaches a maximum of 3 Mbps for video streaming traffic flow in the case of applying the proposed adaptive QoS framework versus a maximum of 2 Mbps without the proposed framework. Moreover, the throughput of VoIP improved to reach 10 Mbps in the case of applying the proposed adaptive QoS framework versus a maximum of 8 Mbps without the proposed framework as depicted in Figure 9. Finally, in case of BE traffic, there is improvement in the throughput as shown in Figure 10. It should be noted that improvement of performance metrics for VoIP traffic and BE traffic occurs even before termination of video streaming sessions at time 30, as video streaming requirements in terms of bandwidth was lower than allocated bandwidth in this scenario.

#### 3.2.3. Scenario 2

**Figure 11:**
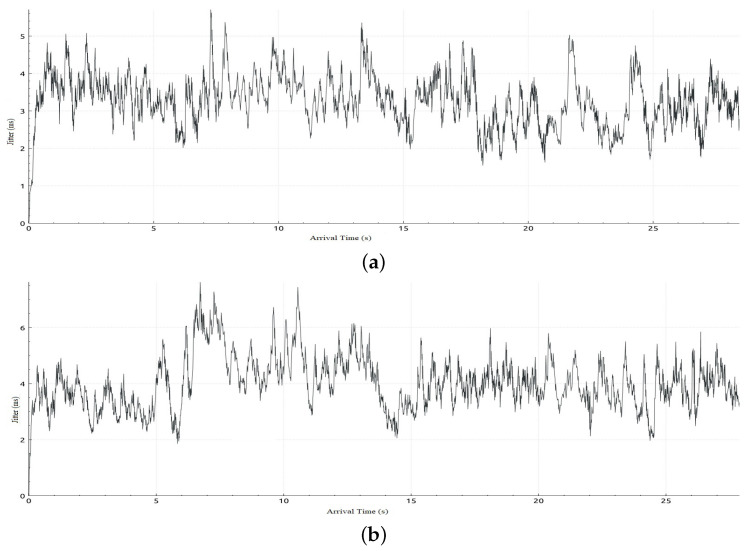
Jitter obtained in Scenario 2 in case of applying and without applying proposed adaptive QoS model for 30-s video streaming traffic flow, (**a**) jitter obtained in Scenario 2 in case of applying proposed model, (**b**) Jitter obtained in Scenario 2 without applying proposed adaptive QoS model.

**Figure 12:**
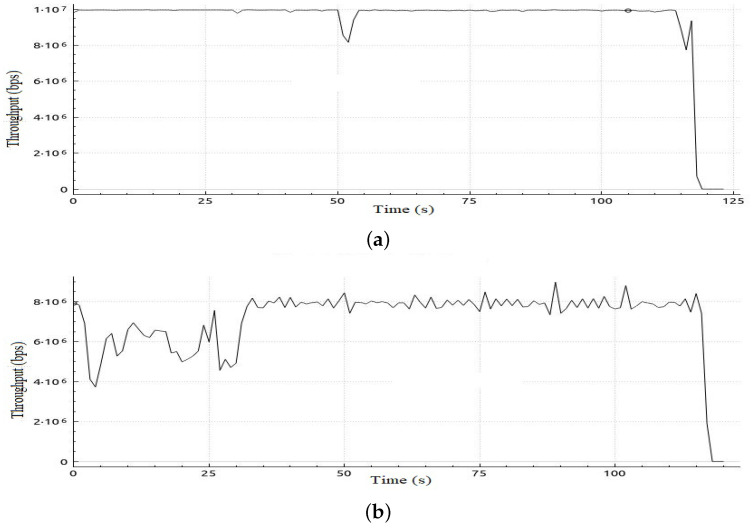
Throughput obtained in Scenario 2 in case of applying and without applying proposed adaptive QoS model for 120-s VoIP traffic flow, (**a**) Throughput obtained in Scenario 2 in case of applying proposed model, (**b**) Throughput obtained in Scenario 2 without applying proposed adaptive QoS model.

**Figure 13:**
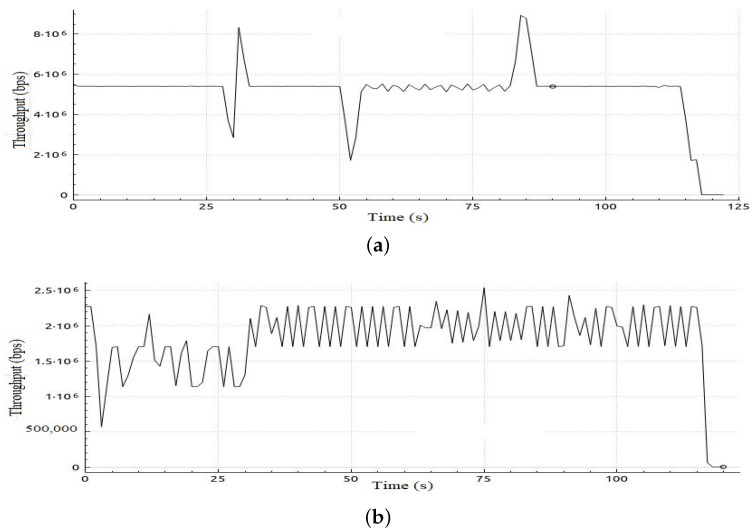
Throughput obtained in Scenario 2 in case of applying and without applying proposed adaptive QoS model for 120-s BE traffic flow, (**a**) Throughput obtained in Scenario 2 in case of applying proposed model, (**b**) Throughput obtained in Scenario 2 without applying proposed adaptive QoS model for 120-seconds BE traffic flow.

In this scenario, we deploy the same topology tested in Scenario 1 with a different initialization phase as defined in Table 9. This scenario aims to investigate the impact of the initial allocation of bandwidth on performance in case it was not in favor of video streaming traffic flow. Table 10 shows sample calculation for the adaptation phase for Scenario 2. In Table 11, a comparison of the results of the jitter and number of lost packets for video streaming traffic flows in both cases, with and without the proposed adaptive QoS framework, is given. As illustrated in Table 11, our proposed adaptive QoS framework decreases the number of lost packets by an average of 7% for video streaming traffic flows and improves the mean jitter for video streaming traffic flows by 14.7% even if the initial allocation was not in favor of the video streaming traffic. This results in reassignment of free bandwidth to the video traffic as it has the highest priority when reassigning free bandwidth. Figure 11 shows the jitter result for video streaming traffic flow. In addition, improvement in throughput for VoIP and BE traffic can be deduced from Figure 12 and Figure 13 respectively. In the case of VoIP average throughput of 9.6 Mbps and 5.2 Mbps in case of BE traffic compared to 7.1 Mbps and 1.8 Mbps with a 26% improvement in the case of VoIP and 64% in case of BE as depicted in Table 12.

#### 3.2.4. Scenario 3

**Figure 14:**
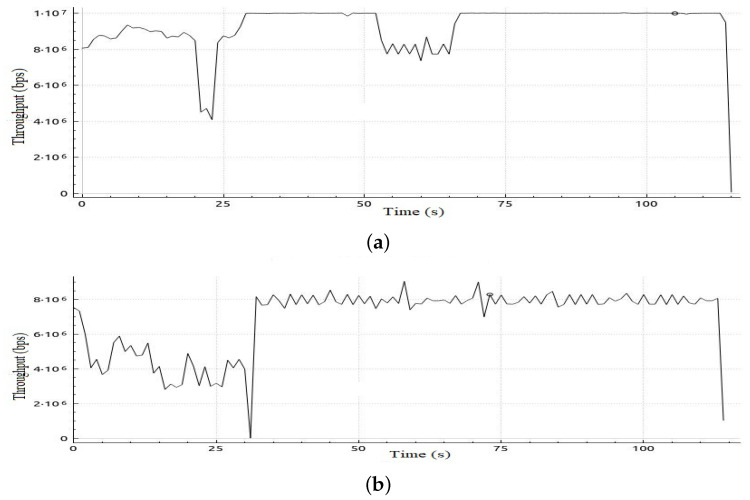
Throughput obtained in Scenario 3 in case of applying and without applying proposed adaptive QoS model for 120-seconds VoIP traffic flow, (**a**) Throughput obtained in Scenario 3 in case of applying proposed model, (**b**) Throughput obtained in Scenario 3 without applying proposed adaptive QoS model.

**Figure 15:**
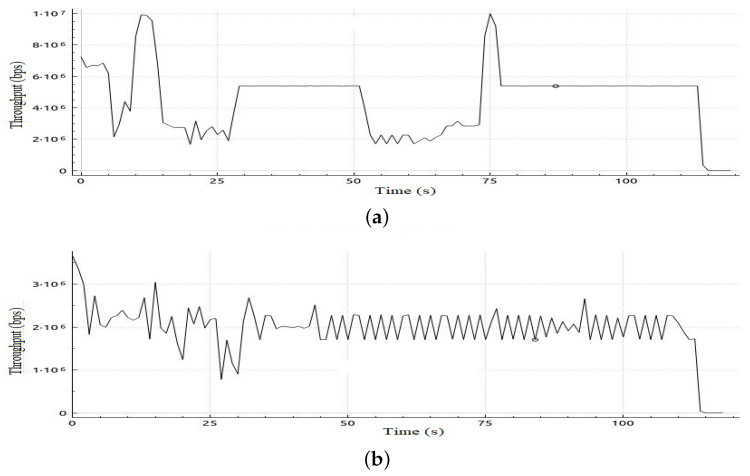
Throughput obtained in Scenario 3 in case of applying and without applying proposed adaptive QoS model for 120-seconds BE traffic flow (**a**) Throughput obtained in Scenario 3 in case of applying proposed model, (**b**) Throughput obtained in Scenario 3 without applying proposed adaptive QoS model.

In this scenario, we investigate the impact of increasing the number of parallel video streaming sessions on the performance of our proposed adaptive QoS framework. In this scenario, we increased the number of parallel video streaming to four. Results revealed an improvement in packet loss, by 32.5% compared to the case without applying the proposed QoS model. Although in this scenario the mean jitter is in favor of the case of not applying the QoS model as shown in Table 13, the higher packet loss percentage makes our proposed model performance higher because the mean jitter, in this case, is calculated on a 32.5% lower number of packets due to this percentage of packet loss. In addition, improvement in throughput for VoIP and BE traffic can be deduced from Figure 14 and Figure 15, respectively. In the case of VoIP, an average throughput of 9.4 Mbps, and 4.6 Mbps in the case of BE traffic was observed, compared to 7 Mbps and 2 Mbps with a 25.5% improvement in the case of VoIP and 56.5%, respectively, in case of BE as depicted in Table 14.

## 4. Conclusions

In this work, we proposed an intelligent adaptive QoS framework to support video streaming in IoT environments. We deployed detecting weapons in video surveillance systems as our case study. The proposed framework integrates deep-learning AI models deployed at the edge with the SDN paradigm to support multimedia traffic constraints. A deep learning-based weapon detection model was presented and evaluated using precision, recall, and mean average precision to detect any keyframes including weapons and, accordingly, the proposed AI model would transmit them. The results showed that the YOLOv5n model outperformed YOLOv5-lite e and YOLOv5-lite s. Evaluation of the proposed adaptive QoS model revealed improvements in performance in terms of jitter, packet loss, and average throughput in all scenarios studied. The improvement was due to several reasons. First, the AI model used at the edge decreases the demand for extensive bandwidth usage due to the reduction of the size of the video surveillance file to be transmitted and sending only the scenes containing weapons. Second, leveraging the SDN paradigm helped the adaptation phase of the algorithm to succeed in all scenarios due to the global view of the network the SDN paradigm offers. In the future, we will investigate the differences between the theoretical and real-world performance of the proposed model. There is also room for improvement of the weapon detection results achieved by the YOLOv5n model while maintaining the real-time constraints. Moreover, in the context of SDN, we will investigate if the placement of SDN controllers and the number of SDN controllers have any impact on the performance.

## Figures and Tables

**Table 1:** Literature Review of SDN-based Video Surveillance Systems.

Proposed Model	Role Of SDN	Performance Gain	Role of AI Techniques Used
SmartArgos [21]	SDN controller informs switches of the chosen k streams to throttle the remaining n-k stream	Conserve wireless bandwidth	At the back-end server to select video streams subset of k streams to display to the monitoring person
Intelligent system for video surveillance in IoT networks [22]	Network core to manage different IoT networks. SDN Controller collects statistics from OpenFlow switches and passes them to the AI module to decide the best action that is propagated back by the controller to switches in case of multimedia traffic	lower jitter and loss rate	AI module for error control in multimedia transmission. Evaluates the resources required and the best action to provide an adequate level of QoE.
Our proposed model	Network core and QoS: adaptive allocation of bandwidth among different traffic flows based on the type of traffic and reassignment of free bandwidth to bandwidth-hungry traffic with priority to video traffic flow class	Higher average throughput, lower jitter, and lower packet loss	AI module available at the edge to filter video file generated by IP-surveillance camera to detect weapons for early detection of crime and to decrease bandwidth requirements by sending crime scenes only

**Table 2:** Testing results of object detection algorithms.

		YOLOv5n			YOLOv5-lite e			YOLOv5-lite s		
Class	Labels	P	R	mAP	P	R	mAP	P	R	mAP
				@0.5			@0.5			@0.5
all	857	0.88	0.80	0.88	0.78	0.79	0.83	0.79	0.83	0.84
pistol	85	0.79	0.95	0.95	0.74	0.88	0.87	0.70	0.92	0.88
smartphone	140	0.95	0.79	0.90	0.94	0.73	0.90	0.91	0.82	0.92
knife	452	0.97	0.86	0.96	0.89	0.87	0.93	0.93	0.90	0.95
purse	71	0.79	0.73	0.77	0.68	0.70	0.75	0.69	0.77	0.70
bill	52	0.85	0.85	0.93	0.85	0.88	0.90	0.85	0.89	0.93
card	57	0.95	0.60	0.76	0.59	0.67	0.61	0.67	0.70	0.66

**Table 3:** Charactersitics of different edge suitable yolo models.

Model	Number of Parameters
YOLOv5-lite e	0.72 M
YOLOv5-lite s	1.55 M
YOLOv5n	1.77 M

**Table 4:** Software versions used in framework evaluation.

Software	Description
Python 2.7.18	Programming Language
Linux	Host OS
VLC 3.0.11.1	Media Player
Open vSwitch 2.13.3	Virtual multilayer switch with OpenFlow support
ubuntu 20.1	Virtual Machine
RYU Controller	SDN Controller
Mininet 2.3.1	Network Emulator
Wireshark 3.2.7	Packet Capture Tool
iperf 2.0.13	Network testing tool

**Table 5:** Scenario 1 Initialization Phase.

Destination Port Number Used for Classification	Defined Classes	Meter-ID	Meter-Band
Dest. Port number 5004	Class 1 Video Streaming Traffic Flow	1	0.5 · BW = 5 Mbps
Dest. Port number 6000	Class 2 VoIP Traffic Flow	2	0.25 · BW = 2.5 Mbps
Dest. Port number 7000	Class 3 Best-Effort Traffic Flow	3	0.25 · BW = 2.5 Mbps

**Table 6:** Scenario 1 Adaptation Phase.

Class-ID	Allocated Bandwidth (ABW)	Required Bandwidth (RBW)	Free Bandwidth
1	5 Mbps	Each video stream requires 1 Mbps	3 Mbps
2	2.5 Mbps	Each VoIP client stream requires 3 Mbps	0 Mbps
3	2.5 Mbps	5 Mbps	0 Mbps

**Table 7:** Scenario 1 Video Streaming Performance Results.

Traffic Flow	Mean Jitter in Case of AQoS	Mean Jitter in Case QoS Not Applied	% of Lost Packets in Case of AQoS	% of Lost Packets in Case of AQoS not Applied
Class 1 Video Streaming	3.6 ms	3.8 ms	0.3%	8.4%
Class 1 Video Streaming	3.5 ms	3.9 ms	0.4%	8.0%

**Table 8:** Scenario 1 Throughtput Results.

Average Throughput	In Case of Adaptive QoS	In Case QoS Not Applied
In case of VoIP	9.2 Mbps	7.1 Mbps
In case of BE	7.4 Mbps	1.8 Mbps

**Table 9:** Scenario 2 Initialization Phase.

Destination Port Number Used for Classification	Defined Classes	Meter-ID	Meter-Band
Dest. Port number 5004	Class 1 Video Streaming Traffic Flow	1	0.1 · BW = 1 Mbps
Dest. Port number 6000	Class 2 VoIP Traffic Flow	2	0.1 · BW =1 Mbps
Dest. Port number 7000	Class 3 Best-Effort Traffic Flow	3	0.8 · BW = 8 Mbps

**Table 10:** Scenario 2 Adaptation Phase.

Class-ID	Configured Bandwidth (CBW)	Required Bandwidth (RBW)	Free Bandwidth
1	1 Mbps	2 Mbps	0 Mbps
2	1 Mbps	Each VoIP client stream requires 3 Mbps	0 Mbps
3	8 Mbps	5 Mbps	3 Mbps

**Table 11:** Scenario 2 Video Streaming Performance Results.

Traffic Flow	Mean Jitter in Case of AQoS	Mean Jitter in Case QoS Not applied	% of Lost Packets in Case of AQoS	% of Lost Packets in Case of AQoS not Applied
Class 1 Video Streaming	3.2 ms	3.9	0	7.2%
Class 1 Video Streaming	3.4 ms	3.8	0	6.9%

**Table 12:** Scenario 2 Throughtput Results.

Average Throughput	In Case of Adaptive QoS	In Case QoS Not Applied
In case of VoIP	9.6 Mbps	7.1 Mbps
In case of BE	5.2 Mbps	1.8 Mbps

**Table 13:** Scenario 3 Performance Results in case of 4-parallel video streaming session.

Mean Jitter in Case of Adaptive QoS	% of Lost Packets in Case of AQoS	Mean Jitter In Case QoS Not Applied	% of Lost Packets in Case of AQoS Not Applied
7.7 ms	12.8%	7.0 ms	18.9%

**Table 14:** Scenario 3 Performance Results.

Average Throughput	In Case of Adaptive QoS	In Case QoS Not Applied
In case of VoIP	9.4 Mbps	7.0 Mbps
In case of BE	4.6 Mbps	2.0 Mbps

## Data Availability

SOHAs weapon dataset: https://github.com/ari-dasci/OD-WeaponDetection/tree/master/Weapons%20and%20similar%20handled%20objects, accessed on: 25 May 2022.

## Abbreviations

The following abbreviations are used in this manuscript:

AI	Artificial Intelligence
BE	Best-effort (BE)
CAGR	Compound Annual Growth Rate
C-DPI	Controller-Data Plane Interface
DiffServ	Differentiated Service
IoMT	Internet of Multimedia Things
ITS	Intelligent Transportation Systems
IoT	Internet of Things
IP	Internet Protocol
IntServ	Integrated Service
M2M	Machine to Machine
OVSDB	Open vSwitch Database
OVS	Open vSwitch
QoS	Quality of Service
QoE	Quality of Experience
REST	REpresentational State Transfer
SDN	Software Defined Networks
SDIoT-Edge	Software-Defined Internet of Things-Edge
SAN	Smart Access Nodes
